# Fluids and barriers of the CNS establish immune privilege by confining immune surveillance to a two-walled *castle moat *surrounding the CNS *castle*

**DOI:** 10.1186/2045-8118-8-4

**Published:** 2011-01-18

**Authors:** Britta Engelhardt, Caroline Coisne

**Affiliations:** 1 Theodor Kocher Institute, University of Bern, CH-3012 Bern, Switzerland

## Abstract

Neuronal activity within the central nervous system (CNS) strictly depends on homeostasis and therefore does not tolerate uncontrolled entry of blood components. It has been generally believed that under normal conditions, the endothelial blood-brain barrier (BBB) and the epithelial blood-cerebrospinal fluid barrier (BCSFB) prevent immune cell entry into the CNS. This view has recently changed when it was realized that activated T cells are able to breach the BBB and the BCSFB to perform immune surveillance of the CNS. Here we propose that the immune privilege of the CNS is established by the specific morphological architecture of its borders resembling that of a medieval castle. The BBB and the BCSFB serve as the *outer walls *of the castle, which can be breached by activated immune cells serving as messengers for outside dangers. Having crossed the BBB or the BCSFB they reach the *castle moat*, namely the cerebrospinal fluid (CSF)-drained leptomeningeal and perivascular spaces of the CNS. Next to the CNS parenchyma, the *castle moat *is bordered by a second *wall*, the *glia limitans*, composed of astrocytic foot processes and a parenchymal basement membrane. Inside the *castle*, that is the CNS parenchyma proper, the *royal family *of sensitive neurons resides with their *servants*, the glial cells. Within the CSF-drained *castle moat*, macrophages serve as guards collecting all the information from within the *castle*, which they can present to the immune-surveying T cells. If in their communication with the *castle moat *macrophages, T cells recognize their specific antigen and see that the *royal family is *in danger, they will become activated and by opening *doors in the outer wall of the castle *allow the entry of additional immune cells into the *castle moat*. From there, immune cells may breach the inner *castle wall *with the aim to defend the *castle inhabitants *by eliminating the invading enemy. If the immune response by unknown mechanisms turns against self, that is the *castle inhabitants*, this may allow for continuous entry of immune cells into the *castle *and lead to the death of the *castle inhabitants*, and finally members of the *royal family*, the neurons. This review will summarize the molecular traffic signals known to allow immune cells to breach the outer and inner *walls *of the CNS *castle moat *and will highlight the importance of the CSF-drained *castle moat *in maintaining immune surveillance and in mounting immune responses in the CNS.

## Introduction

Traditionally, the central nervous system (CNS) was viewed as an immunologically-privileged site, which was interpreted as the complete absence of immune surveillance of this tissue [1]. The theoretical basis of these considerations were that CNS homeostasis, which is required for the proper communication of neurons, would not tolerate routine immune cell patrolling in their search for relevant antigens. Experimental findings supporting this notion were that allo- and xenogenic (from different species) tissue grafts, when transplanted into the CNS are much less efficiently rejected by the recipient when compared to transplantation to orthotopic (original) sites. Additionally, the CNS parenchyma is devoid of cells constitutively expressing MHC class I and II and therefore the molecules required by T cells to recognize their antigen. In addition, the CNS lacks lymphatic vessels and thus the commonly established pathways of the afferent communication arm of the immune system. Finally, it was thought that the efferent arm of the immune system to the CNS was completely blocked by the endothelial blood-brain barrier (BBB) and the epithelial blood-cerebrospinal fluid barrier (BCSFB) establishing the barriers between the changing blood milieu and the CNS. The view of immunological ignorance of the CNS has, however, been in conflict with observations by Medawar and colleagues that an allogenic tissue graft into the brain, which would be tolerated in a naive host, was readily rejected in a recipient which was sensitized to the allo-antigens before the transplantation [2]. These observations suggested that T cells activated outside the CNS found a way across the brain-barriers and mounted an immune response within the CNS. Subsequent observations in a number of immune-mediated CNS pathologies including chronic inflammatory diseases such as multiple sclerosis and its animal model, experimental autoimmune encephalomyelitis (EAE), made it obvious that the view of immune privilege for the CNS as the absence of immune surveillance, was in fact too far reaching. In contrast to multiple sclerosis, the etiology of EAE is well established as is it induced by the intravenous transfer of activated neuro-antigen-specific T cell blasts or by subcutaneous immunization with myelin antigens in susceptible animals. As induction of EAE does not require any manipulation of the CNS proper, this animal model has proven to be very valuable for studying immune cell entry into the CNS. By studying EAE we have learned that activated T cells can indeed migrate across either the BBB or the BCSFB in the absence of CNS inflammation and enter the cerebrospinal fluid (CSF)-drained leptomeningeal and perivascular spaces. Therefore we propose that the immune privilege of the CNS is established by a unique architecture resembling that of a medieval castle. In this view the CSF space resembles the *castle moat *of a medieval *castle*, which is bordered at the outside by the BBB and the BCSFB - the outer *castle wall*- and towards the inside by the *glia limitans *- the inner *castle wall*. Inside the *castle*, that is the CNS parenchyma proper, the *royal family *of sensitive neurons resides with their *servants*, the glial cells. Immune surveillance is restricted to the *castle moat*, where perivascular and meningeal macrophages serve as guards that continuously collect information from within the *castle*, and present this information to the *messengers*, the T cells. If in their communication with the *castle moat *guards, T cells recognize their antigen they will become activated and open *gates *in the outer *castle wall *allowing the entry of more immune cells from outside. Within the *castle moat *the immune cells then mount an invasion of the *castle*, breaching the inner *castle wall *and entering the *castle *with the aim to eliminate any intruders. If for reasons unknown, T cells turn their attack to the inhabitants of the *castle*, chronic immune cell invasion of the *castle *will eventually kill many of the *castle *inhabitants leading to neuronal deficits as observed in chronic neuroinflammatory diseases of the CNS such as multiple sclerosis [3].

## Architecture of the brain barriers: the *castle walls *and the *castle moat*

Brain barriers are composed of the endothelial blood-brain barrier (BBB) and the epithelial blood-cerebrospinal fluid barrier (BCSFB), which protect the CNS from the changing milieu of the periphery. The BBB is formed by highly-specialized endothelial cells that tightly restrict the paracellular and transcellular passage of hydrophilic molecules into the CNS by an elaborate network of complex tight junctions between the endothelial cells, in conjunction with a lack of fenestrations and low pinocytotic activity [4]. To fulfill the high metabolic requirements of the CNS parenchyma, sophisticated transport mechanisms operate in BBB endothelial cells ensuring the transport of nutrients from the blood into the CNS and the rapid extrusion of toxic metabolites out of the CNS [5].

Besides the BBB, epithelial cells of the choroid plexus establish the BCSFB, taking over similar functions as the BBB. The choroid plexus, which secretes the cerebrospinal fluid (CSF), consists of villous structures that extend from the ventricular surfaces into the lumen of the ventricles. It is formed by an extensive network of fenestrated capillaries, enclosed by a monolayer of cuboidal epithelium. In contrast to the CNS parenchyma, where microvessels establish the BBB, the microvessels within the choroid plexus stroma are fenestrated allowing the free diffusion of solutes between the blood and the choroid plexus parenchyma through inter-endothelial gaps (reviewed in [6]). The actual barrier that inhibits free diffusion of water-soluble molecules between the bloodstream and the CNS is formed by the choroid plexus epithelial cells that display unique parallel tight junctions [7,8]. Again, similar to the BBB, the barrier and secretory functions of the choroid plexus epithelial cells are maintained by the polarized expression of a number of specific transmembrane transport systems that allow directed transportation into the CSF and the removal of toxic agents out of the CSF [6].

Proper function of the BBB has been shown to rely critically on the sustained interaction with the adjacent extracellular matrix components and cellular elements [9]. The fully-differentiated BBB has therefore recently been referred to as the neurovascular unit (NVU). Within the NVU the highly specialized endothelial cells are the basis of the BBB, which is supported by an underlying endothelial basement membrane embedding a high number of pericytes [10,11]. A layer of astrocytic endfeet along with their parenchymal basement membrane are referred to as the *glia limitans perivascularis *[4,12]. The *glia limitans *and the BBB delineate a CSF-filled space, in which host antigen-presenting cells are localized [13-15]. At the level of parenchymal vessels the perivascular space is narrow. The CSF within these perivascular spaces drains into the subarachnoid space on the surface of the brain and the spinal cord [16]. There the CSF space widens as the meningeal blood vessels lack the direct ensheathment by the *glia limitans perivascularis *[17]. However, the CSF-drained subarachnoid space is also sealed off towards the neuropil by the *glia limitans superficialis*, which resembles the *glia limitans perivascularis *and surrounds the entire surface of the brain and spinal cord [13]. Nevertheless, meningeal CNS microvessels also establish a functional BBB [18], although subtle differences exist between the parenchymal and meningeal BBB, with parenchymal but not meningeal endothelial cells lacking storage of certain adhesion molecules in their Weibel Palade bodies [19,20], suggesting that at the level of the meninges the BBB might provide some additional cues for immune cells to reach the CSF-drained leptomeningeal spaces.

In contrast to the BBB, much less is known about how barrier characteristics of the BCSFB are maintained but it is reasonable to assume that the CSF provides signals for BCSFB maintenance. Cells found in tight association with the apical aspect of the BCSFB have been described as Kolmer cells or epiplexus cells and are most probably antigen-presenting cells (reviewed in [8]).

## Immune surveillance of the CNS: lessons from experimental autoimmune encephalomyelitis (EAE)

Direct evidence for T cell trafficking into the healthy CNS came from tracing radioactively-labeled encephalitogenic CD4^+ ^T cell blasts infused into the circulation of Lewis rat that resulted in the development of experimental autoimmune encephalomyelitis (EAE), considered to be the best animal model for multiple sclerosis (MS) (reviewed by [21]).

MS and EAE are characterized by a massive infiltration of circulating immune cells into the CNS, resulting in central inflammation, oedema formation and demyelination, the basis for the onset of the clinical manifestations of these diseases. Both diseases show clinical and histopathological similarities, however, whereas MS etiology remains unclear and shows a great variety of neuropathological changes, EAE is a T cell-mediated autoimmune inflammatory disease with a well-described CNS pathology. The model disease of EAE can be generated either upon immunization of susceptible naive recipients with CNS myelin antigens or by intravenous injection of freshly-activated - but not resting - CNS myelin-specific CD4^+ ^T cell subsets [22], supporting the concept that only activated T cells can breach the BBB or BCSFB to reach the CSF-filled spaces, where upon encounter of the antigen-presenting cells they can trigger the molecular events leading to the development of the clinical manifestations of the disease.

It has therefore been recognized that the interaction of CD4^+ ^T lymphoblasts with the healthy brain barriers represents the critical step in the initiation of the disease. Few studies have, however, directly addressed the molecular mechanisms of T lymphoblast migration across the BBB. We have approached this question by means of intravital microscopy (IVM) of the spinal cord, a technique that allowed us to directly observe the interaction of circulating fluorescently-labeled encephalitogenic T cell blasts with the spinal cord microvasculature [23]. Using this technique, our laboratory demonstrated that freshly-activated encephalitogenic Th1 cell blasts are able to interact with spinal cord capillaries and post-capillary venules in healthy animals [23]. Th1 cell interaction with the non-inflamed BBB occurred in post-capillary venules and was found to be unique due to a lack of rolling. Rather, encephalitogenic Th1 cell blasts were abruptly captured to the spinal cord post-capillary venule wall, followed by firm adhesion of the Th1 cell to the venule [23]. Both capture and firm adhesion were found to be mediated through the interaction of α4-integrins on the Th1 cell with endothelial VCAM-1, whereas diapedesis of Th1 cells across the venule wall was found to be supported by the αLβ2-integrin (leukocyte function-associated antigen (LFA)-1) [24]. However, successful diapedesis of Th1 cells into the perivascular space across the intact BBB was only observed at 4 to 6 hours post-injection [24] suggesting that breaching the healthy BBB remains difficult. Although we observed α4-integrin/VCAM-1-dependent capillary plugging of Th1 cells, Th1 cell diapedesis was solely observed at the level of spinal cord post-capillary venules. In fact, IVM studies of T cell interaction with the non-inflamed blood-retinal barrier, which displays tight junctions and barrier characteristics comparable to the BBB, provided similar observations [25]. T cell blasts were also observed to abruptly stop without prior rolling within non-inflamed retinal microvessels and to take up to 16 hours to penetrate the vascular wall. These authors found that successful diapedesis of T cell blasts across the blood-retinal barrier required local activation of endothelial cells as demonstrated by the upregulation of the intercellular adhesion molecule-1 (ICAM-1) on the endothelial surface that is required for T cell diapedesis across vascular barriers [25]. Thus, T lymphoblast entry across the parenchymal BBB seems to be a difficult task and only few T cells seem to reach the perivascular space, which raises the question whether T lymphoblast extravasation across the BBB would be sufficient to ensure the immune surveillance of the entire CNS.

Therefore, other routes of T cell entry into the healthy CNS need to be considered. In fact, *in vivo *homing studies following fluorescently labeled encephalitogenic T cell blasts, showed that two hours following their systemic injection these cells were already found within the leptomeningeal compartment [26]. The molecular key allowing for T cell entry into this compartment was defined to be P-selectin, which is stored in Weibel-Palade bodies in endothelial cells of leptomeningeal, but not parenchymal BBB, endothelial cells [27,28]. Thus, P-selectin seems to provide easier access for T cell blasts into the leptomeningeal space, whereas in the absence of P-selectin at the level of the parenchymal BBB, entry of T cells into the narrow perivascular space is less efficient as it takes longer and requires different molecular keys as outlined above. T cells, which have entered the CSF-drained subarachnoid space, where they perform immune surveillance of the CNS-drained leptomeningeal space, start to screen leptomeningeal macrophages for their specific antigen. Recently, two-photon imaging studies of the rat spinal cord microvasculature from Bartholomäus and colleagues confirmed that T cell blasts preferentially enter the leptomeningeal spaces [29]. However, without any specific antigen-triggering, activated T cells were restricted to the leptomeningeal space, as they did not manage to make their way through the *glia limitans *into the CNS parenchyma [29,30].

Following fluorescently-labeled encephalitogenic T cell blasts *in vivo *also detected T cells within the choroid plexus parenchyma two hours following their systemic injection [26]. Similar to the meningeal BBB, P-selectin was found to be expressed on the fenestrated choroid plexus capillaries [31], suggesting that again P-selectin may specifically guide T cell blasts into the choroid plexus. However, T cells that have crossed the choroid plexus microvasculature are localized within the choroid plexus stroma and thus still outside of the CNS. To get access to the CSF drained space these T cells still need to penetrate the epithelial BCSFB. The observation that in humans high numbers of central memory T cells are present within the CSF compartment, when compared to the peripheral blood of healthy individuals, strongly suggests that T cells enter the CSF compartment directly via the choroid plexus across the BCSFB [31,32]. In fact the first molecular explanation of T cell migration across the BCSFB has been recently provided, when studying the role of Th17 cells in the pathogenesis of EAE. Mice deficient for the chemokine receptor CCR6, which is specifically expressed on the Th17 subset of T cells, were found to be resistant to EAE [33]. In this context, CCR6^-/- ^immune cells were found to have accumulated within the choroid plexus parenchyma, where the chemokine CCL20, the ligand for CCR6, is constitutively produced by the choroid plexus epithelium. CCL20 is not expressed at the BBB and CCR6 was not involved in T cell interaction with the BBB. As CCR6^-/- ^mice developed EAE following the transfer of a subclinical number of fluorescently-labelled myelin-specific Th17 cells expressing CCR6, it was concluded that EAE is initiated by the CCR6/CCL20-dependent migration of Th17 cells across the non-inflamed BCSFB. Additional molecular mechanisms of T cell migration across the BCSFB remain to be investigated. Although the choroid plexus epithelium constitutively expresses ICAM-1 and VCAM-1, these molecules are expressed in a highly polarized fashion at the apical side of the choroid plexus epithelium [34,35] and are therefore not accessible to T cells migrating across the BCSFB from the basolateral to the apical side. Once they have penetrated the BCSFB, Th17 blasts seem to rapidly travel to the leptomeningeal spaces, where fluorescently-labelled CCR6^+ ^Th17 cells were exclusively found within the leptomeningeal compartment [33].

Taken together, these observations demonstrate that immune surveillance of the CNS is ensured by allowing activated immune cells access *via *the BBB and the BCSFB to the CSF space, where they can interact with the antigen presenting cells. In the absence of antigen recognition these T cell blasts remain confined to the CSF drained spaces and therefore do not impair CNS homeostasis. However, if they recognize their specific antigen, these immune cells will mount an inflammatory response that brings additional immune cells from the periphery into the perivascular spaces and from there they eventually breach the *glia limitans*, to mount an immune defence within the CNS parenchyma.

## T cell invasion in neuroinflammation: Lessons from experimental autoimmune encephalomyelitis (EAE)

Reactivation of the T cells, by their antigen-presenting cells bearing the cognate antigen, within the CSF-filled CNS spaces will lead to inflammatory changes of the BBB and probably also of the BCSFB. This is achieved by the expression of additional trafficking signals on these barriers allowing the recruitment of numerous inflammatory cells from the bloodstream into the CSF-drained perivascular and subarachnoid spaces. The molecular mechanisms involved in T cell migration across the inflamed BBB have been well studied using EAE, the animal model of multiple sclerosis, and will be summarized here.

## Breaching the BBB - the outer *castle wall*

A number of intravital microscopic studies observing the interaction of myelin-specific T cells with inflamed leptomeningeal brain microvessels in EAE have shown that endothelial P-selectin mediated the rolling of T cells via their selectin ligand PSGL-1 in leptomeningeal post-capillary venules [36-39]. Surprisingly though, numerous studies by us and others demonstrated that blocking or absence of PSGL-1 or E- and P-selectin did not have any significant impact on the recruitment of inflammatory cells into the CNS and failed to affect the development of the disease [28,38,40-43]. At the same time, initial interaction of T cells with the inflamed BBB is no longer solely dependent on α4β1-integrins, because functional blockade of α4-integrins on human T cells or the deletion of the β1-integrin sub-units on mouse T cells, did not show any impact in the initial interaction of these T cells with the inflamed spinal cord microvasculature of mice afflicted with EAE [44,45]. Because combined blockage of P-selectin and α4-integrins resulted in a more complete inhibition of T cell rolling along inflamed leptomeningeal vessels and produced a greater delay of EAE onset than solely blocking α4-integrins, it is concluded that P-selectin/PSGL-1 and α4-integrins/VCAM-1 interactions might mediate T cell rolling in an overlapping or partially redundant fashion [38].

Firm arrest of T cells at the inflamed post-capillary venule BBB similarly requires signalling via G-protein-coupled receptors present on the surface of the circulating immune cells, as at the healthy BBB [36]. Thus chemokines or eicosanoids displayed on the luminal side of the BBB are involved in inflammatory cell recruitment across the BBB during EAE. Although numerous chemokines have been shown to be involved in EAE pathogenesis [46,47], the ones mediating T cell arrest at the BBB remain to be defined. To date, relatively few chemokines have been reported to be expressed by the BBB endothelium *per se*. These are the lymphoid chemokines CCL19 [48-50] and CCL21 that have been shown to enable the adhesion of encephalitogenic CCR7^+ ^T cells to inflamed brain microvessels *in vitro *[48]. Endothelial expression of CXCL12 is upregulated during EAE, its increased expression may, however, be more related to anti-inflammatory effects [51]. Also, CXCL12 expression is restricted to the basolateral side of the CNS microvascular endothelium, thus facing towards the perivascular space and suggesting a function in this compartment, rather than in T cell recruitment across the BBB. In fact, this chemokine was shown to cause retention of the inflammatory cells expressing CXCR4 that have infiltrated the perivascular space [51]. Taken together, the chemokines involved in T cell migration across the BBB remain to be defined.

G-protein-mediated activation of integrins on the T cells leads to their arrest at the post-capillary venule BBB. Several studies reported the upregulation of the integrin ligands ICAM-1 and VCAM-1 on CNS endothelia [36,52-54] as well as on the choroid plexus epithelium [53] during EAE. In animals afflicted with EAE, the CNS inflammatory cells localized around ICAM-1 and VCAM-1-positive venules stain positive for LFA-1 and α4β1-integrins, the ligands for ICAM-1 and VCAM-1, respectively. In MS lesions, the upregulation of ICAM-1 expression was observed on inflamed vessels [55,56] along with LFA-1-positive staining of most infiltrating immune cells [56]. In contrast to EAE, VCAM-1 expression was described on CNS microvessels in some [57] but not in other [58] MS lesions. However, in human CNS microvessels, the CS1 domain of a spliced variant of fibronectin (FN-CS1) could serve as an alternative ligand for α4-integrin [59].

Numerous *in vitro *studies have demonstrated that under static conditions T cells bind via LFA-1 and α4-integrins to their respective endothelial ligands, ICAM-1 and VCAM-1, on inflamed cerebral vessels in a Stamper-Woodruff assay [53,60] or on cultured brain microvascular endothelial cells [59,61-64]. Recent elegant *in vitro *and *in vivo *studies have specified that shear-resistant T cell arrest on the inflamed BBB is mediated by LFA-1/ICAM-1 and α4β1-integrin/VCAM-1 interactions [29,45,65].

The development of EAE can be abrogated by blocking α4-integrins or VCAM-1 as shown by many laboratories [54,60,66-68]. The therapeutic effect of these blocking antibodies leads to the inability of the immune cells to breach the BBB and has been successfully translated into the clinic, where the humanized monoclonal antibody against α4-integrins, natalizumab is successfully used to treat relapsing-remitting MS (summarized in [69]). We recently demonstrated by means of intravital microscopy (IVM) that natalizumab specifically inhibits the adhesion, but not the rolling or capture, of human T cells with the inflamed spinal cord microvasculature in mice with acute EAE [44], providing the first direct *in vivo *proof of concept of this therapy in MS.

Despite the proven contribution of LFA-1 and its endothelial ligands ICAM-1 and ICAM-2 in T cell arrest on the inflamed BBB endothelium, their functional inhibition or absence in a variety of EAE models produced contradictory results, ranging from inhibiting EAE to increasing severity of EAE or having no effect at all [70-72]. Therefore, further investigations will be necessary to fully delineate the role of LFA-1 and its ligands ICAM-1 and ICAM-2 beyond their mere contribution to T cell trafficking across the BBB.

Recent elegant *in vitro *and *in vivo *studies demonstrated that once T cells have arrested on the BBB surface, T cells get polarized and start to crawl on the endothelium against the direction of the blood flow until they find sites permissive for diapedesis [29,65]. T cell polarization and crawling is mediated by LFA-1 binding to endothelial ICAM-1 and ICAM-2 with no further contribution of α4-integrin/VCAM-1 [61,65,73]. Binding of LFA-1 to endothelial ICAM-1 was shown to trigger signaling cascades in brain endothelial cells leading to the activation of the tyrosine kinase p60, phosphorylation of cortactin, to intracellular calcium increase and RhoA activation, all triggering the reorganization of the endothelial actin-cytoskeleton (summarized in [74]). Nevertheless, although diapedesis of T cells across the BBB seems to be more efficient in the presence of ICAM-1 and ICAM-2 [75], passage of T cells can be observed in the absence of ICAM-1 and ICAM-2, suggesting that additional molecular cues can be used by T cells for diapedesis across the BBB, or that alternative pathways exist. We and others who have investigated leukocyte diapedesis across the BBB into the inflamed CNS by means of transmission electron microscopy have invariably observed leukocyte migration to occur through the endothelial cells, i.e. via a transcellular pathway, leaving the tight junctions intact [76-81]. However, other researchers studying T cell diapedesis across the BBB *in vitro *have doubted this view and support the view that paracellular diapedesis occurs through the BBB tight junctions (summarized in [76]). At present, the molecular mechanisms guiding transcellular or paracellular diapedesis of T cells across the inflamed BBB are not well understood and have rarely been specifically addressed in the context of the BBB. One exception is the endothelial junctional molecule PECAM-1. PECAM-1^-/- ^mice show increased infiltration of immune cells across the BBB along with an enhanced vascular permeability [82] demonstrating that impairment of endothelial junctions at the BBB might favor a paracellular pathway for T cell diapedesis. Thus, the molecular mechanisms of the multistep recruitment of T cells across the inflamed BBB are not yet fully understood and the role of additional molecules such as JAM-A [83] or ALCAM [84], which have been suggested to be involved in these processes need to be explored in more detail.

Following their diapedesis across the inflamed BBB, T cells need to make their way across the acellular extracellular matrix components of the endothelial basement membrane. The endothelial basement membrane consists of a number of glycoproteins such as laminins, collagen type IV, nidogens, and heparan sulfate proteoglycans. Recently, it has been observed that T cell extravasation at the BBB occurs preferentially at sites, where the endothelial basement membrane contains laminin α4 rather than laminin α5 [12,85]. In laminin α4-deficient mice, compensatory expression of laminin α5 is found in the endothelial cell basement membranes of the CNS and in these mice, EAE progression was ameliorated in association with a reduction of cellular infiltrates [85]. These findings suggest that laminin α5 does not support T cell migration across the BBB basement membrane and may even redirect T cells to specific sites devoid of this laminin isoform, where α6β1-integrin/laminin α4 binding will facilitate the transmigration of T lymphocytes across the endothelial basement membrane into the perivascular space [85].

In contrast to the BBB, the molecular mechanisms for T cell diapedesis across the BCSFB during EAE have not been investigated. During EAE, upregulated expression of functional ICAM-1 and VCAM-1 and induction of MAdCAM-1 is observed [34]. As these molecules are exclusively expressed on the apical side of the choroid plexus epithelium [35], they are not accessible for T cells crossing the BCSFB from the basolateral to apical sides. Also, in most neuroinflammatory disorders, accumulation of immune cells in the choroid plexus cannot be observed suggesting that this entry port may be more relevant during immune surveillance.

## Immune cells in the CSF-drained fluid spaces - the *castle moat*

Once the endothelial basement membrane has been breached, extravasating encephalitogenic T cells reach the perivascular space, where they have to re-encounter their cognate antigen presented by antigen-presenting cells, in the context of major histocompatibility complex (MHC) class II, in order to pursue their route into the CNS parenchyma. This was demonstrated by the study from Greter and colleagues, in which the clinical development of EAE was shown to be triggered by myelin-reactive T cells following antigen priming by perivascular CD11c^+ ^dendritic cells [15]. A recent elegant two-photon *in vivo *imaging study demonstrated that T cells interact with many leptomeningeal macrophages in search of their specific antigen [29]. In the absence of their specific antigen, T cells were confined to this space, whereas upon recognition of their specific antigen they could invade the CNS parenchyma.

In EAE it has been demonstrated that the typical perivascular inflammatory cuffs are localized between the outer endothelial and inner parenchymal basement membranes, and therefore remain within the perivascular space. Interestingly, clinical signs of EAE only start following the penetration of inflammatory cells across the *glia limitans *and their entry into CNS parenchyma [86].

## Attack of the CNS requires breaching of the *glia limitans*, the *inner wall of the castle*

The CSF-drained perivascular and subarachnoid spaces are sealed off towards the neuropil by the *glia limitans perivascularis *and *glia limitans superficialis*, respectively, surrounding the entire surface of the brain and spinal cord [13]. The *glia limitans *is composed of the parenchymal basement membrane, which is molecularly distinct from the endothelial basement membrane with deposition of laminins α1 and α2 but not the endothelial laminins α4 and α5 (summarized in [9]). The parenchymal basement membrane is laid down by astrocytes which with their foot processes, embrace the entire surface of the CNS parenchyma.

In contrast to the penetration of the BBB, penetration of the *glial limitans *by inflammatory cells requires the activity of matrix metalloproteinases (MMP-2 and MMP-9), which is probably due to the distinct biochemical composition of the parenchymal basement membrane and the tight seal with astrocyte foot processes [86]. Obviously, MMP-2 and MMP-9 are produced by the myeloid cells surrounding the T cells in the perivascular space [86] and allow immune cells to breach the *glia limitans *and reach the CNS parenchyma where they start their attack on the CNS parenchymal cells. Once immune cells have reached the CNS parenchyma the immune attack starts and CNS parenchymal cells may be sacrificed in order to fight the potential infection. In EAE the autoimmune response leads to chronic attack of oligodendrocytes and neurons eventually leading to demyelination and axonal loss, that is to say ultimately the loss of CNS neurons.

## Conclusion

Originally the CNS was considered an immune-privileged organ in the sense that it is ignored by the immune system. Recent studies on T cell trafficking to the CNS in the context of EAE have identified anatomical routes and molecular mechanisms of T cell entry into the CNS during health and disease and suggest that CNS immune privilege is accomplished by the unique architecture of a *medieval castle *that is surrounded by a *two-walled moat*. Activated memory/effector but not resting T cells, are in possession of the molecular keys to breach the *outer castle wall *- the BBB or the BCSFB - without impairing its resistance to assault. Behind this outer cellular barrier, T cells enter the CSF drained ventricular, perivascular and subarachnoidal spaces, which are bordered by two biochemically distinct basement membranes. Immune-surveillance of the CNS is confined to this space, which resembles the castle moat bordered by outer and inner walls and patrolled by guards, the perivascular antigen-presenting cells and the epiplexus cells. If T cells encounter their specific antigen within the CSF-drained *castle moat*, they become activated and will trigger an inflammatory response that leads to the upregulation of novel traffic signals on the BBB and the BCSFB - i.e. opening doors in the outer wall and leading to the recruitment of additional inflammatory cells across this outer barrier. Production of cytokines and proteases allows the degradation of the *glia limitans *- the *inner wall of the castle moat *- and in a second step by lowering the *draw-bridge *across the *castle moat*, large number of infiltrating inflammatory cells will then enter the CNS parenchyma, leading to the cellular assault of the *castle*, respectively. Taken together, it has been realized that the CSF-drained fluid spaces play a central role in establishing CNS immune surveillance and therefore in maintaining CNS immune privilege. Whereas our understanding of the traffic signals involved in T cell migration across the BBB has increased enormously, T cell entry across the BCSFB and the following steps of immune cell entry across the basement membranes and the *glia limitans *remain to be investigated.

## Competing interests

The authors declare that they have no competing interests.

## Authors' contributions

BE developed the basic concept of the review and CC completed the paragraphs. Final editing of the review was done by BE. Both authors have read and approved the manuscript.
